# BECN1 promotes the migration of NSCLC cells through regulating the ubiquitination of Vimentin

**DOI:** 10.1080/19336918.2019.1638690

**Published:** 2019-07-05

**Authors:** Zhujun Cheng, Hongbo Xin, Tianyu Han

**Affiliations:** a Jiangxi Institute of Respiratory Disease, The First Affiliated Hospital of Nanchang University, Nanchang, P.R. China; b Department of Burn, The First Affiliated Hospital of Nanchang University, Nanchang, P.R. China; c The National Engineering Research Center for Bioengineering Drugs and the Technologies, Institute of Translational Medicine, Nanchang University, Nanchang, P.R. China

**Keywords:** BECN1, migration, NSCLC, ubiquitination, Vimentin

## Abstract

BECN1/Beclin1 is one of the key proteins in autophagy regulation. However, the biological functions of BECN1 in non-small cell lung cancer (NSCLC) were obscure. Here, we found that neither BECN1 knockdown nor overexpression affected the proliferation of NSCLC cells. Surprisingly, BECN1 overexpression increased cell migration and knocking down BECN1 significantly reduced the migratory ability of NSCLC cells. We further demonstrated that BECN1 could interact with Vimentin and affected its K48-linked ubiquitination. What’s more, BECN1 could also interact with ubiquitin-specific peptidase 14 (USP14), the key de-ubiquitinase of Vimentin, and regulated USP14 mediated de-ubiquitination of Vimentin. Thus, our studies revealed an oncosupportive role of BECN1 in the migration of NSCLC cells through regulating the ubiquitination of Vimentin.

## Introduction

Autophagy is a multi-step, lysosomal degradation pathway that sequestrates cytoplasmic components for degradation [1,2]. BECN1 is the key protein that assembles cofactors for the formation of BECN1-Vps34-Vps15 complex to trigger a cascade of proteins involved in autophagolysosome formation [3]. The dysfunction of BECN1 has been suggested in many diseases, including cancer [4]. The connection between BECN1 and cancer was first discovered in 1999, and now BECN1 is commonly known as a haploid-insufficient tumor suppressor [5]. Monoallelic deletion of BECN1 gene was reported in 40–70% of ovarian, breast and prostate cancers [6,7]. In mouse models, monoallelic deletion of BECN1 gene showed a significant increase in the number of spontaneous liver and lung cancers, leukemias and lymphomas compared with animals having both alleles [8]. However, there were studies demonstrated that BECN1 might also have oncogenic role. High expression of BECN1 has been reported in patient-derived samples of gastric and colon cancer [9]. In ovarian cancer, knocking down BECN1 decreased cell viability [10]. In breast cancer, BECN1 was required for tumorigenicity of cancer stem cells [11]. These studies demonstrated that the functions of BECN1 in cancer cells were complicated and more studies needed to be done to fully clarify the function of BECN1.

Until now, the functions of BECN1 in NSCLC cells were obscure. Previous studies demonstrated that the expression of BECN1 was significantly reduced in NSCLC tissues compared with the peripheral normal tissues and the reduced expression of BECN1 was associated with poor prognosis [12,13]. However, another study demonstrated that BECN1 showed no association with survival in NSCLC [14]. In A549 cells, knocking down BECN1 promoted cell proliferation and decreased apoptosis [15]. Introduction of BECN1 to the lungs of K-ras (LA1) mice reduced the numbers of tumor on the surface and histopathological tumor progression in the lungs of K-ras (LA1) mice [16]. However, the exactly biological function of BECN1 in NSCLC cells was still unclear. In our present study, we found that neither BECN1 knockdown nor overexpression affected the proliferation of NSCLC cells. However, overexpression of BECN1 remarkably enhanced the migration of NSCLC cells while BECN1 knockdown reduced the migratory ability. We further discovered that BECN1 interacted with Vimentin, which was implicated in epithelial–mesenchymal transition (EMT) and cancer cell migration. The expression of BECN1 could regulate Vimentin expression through affecting USP14 mediated de-ubiquitination of Vimentin. Thus, our study demonstrated that BECN1 could promote cancer progression through facilitating cancer cell migration in NSCLC cells.

## Results

### The expression of BECN1 does not significantly affect the proliferation of NSCLC cells

**Figure 1.**
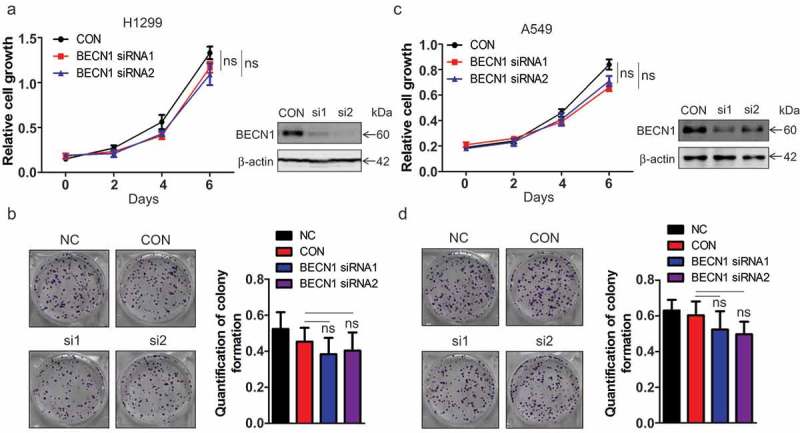
*BECN1 knockdown does not significantly affect the proliferation of NSCLC cells*. (a) H1299 cells were transfected with siRNAs specific for BECN1. Twenty-four hours later, cell growth assay was performed. At the indicated times, cells were fixed with 4% formaldehyde and then stained with 2% crystal violet. The dyes were finally dissolved by 10% acetic acid and the relative proliferation was determined by the absorbance at 595nm. Data represent the average of three independent experiments. ns, *P >*0.05 (Left panel). The knockdown efficiency of the siRNAs targeting BECN1 was determined by Western blot using anti-BECN1 antibody (Right panel). (b) H1299 cells were transfected with BECN1 siRNAs for 24 h and colony formation assay was performed. After ten days, the cells were fixed with 4% formaldehyde and then stained with 2% crystal violet. The colonies were photographed (Left panel). The dyes were dissolved by 10% acetic acid and the quantification of colony formation was determined by the absorbance at 595nm. Data represent the average of three independent experiments. ns, *P >*0.05 (Right panel). (c) A549 cells were transfected with BECN1 siRNAs. Twenty-four hours later, cell growth assay was performed. At the indicated times, cells were fixed with 4% formaldehyde and then stained with 2% crystal violet. The dyes were finally dissolved by 10% acetic acid and the relative proliferation was determined by the absorbance at 595nm. Data represent the average of three independent experiments. ns, *P >*0.05 (Left panel). The knockdown efficiency of the siRNAs targeting BECN1 was determined by Western blot using anti-BECN1 antibody (Right panel). (d) A549 cells were transfected with BECN1 siRNAs for 24 h and colony formation assay was performed. After ten days, the cells were fixed with 4% formaldehyde and then stained with 2% crystal violet. The colonies were photographed (Left panel). The dyes were dissolved by 10% acetic acid and the quantification of colony formation was determined by the absorbance at 595nm. Data represent the average of three independent experiments. ns, *P >*0.05 (Right panel).

BECN1 was first known as a tumor suppressor in breast cancer [5] and the expression of BECN1 was reported to be downregulated in many cancers [12,17–20]. Previous study demonstrated that knocking down BECN1 in A549 cells promoted cell growth and inhibited apoptosis [15]. However, another study demonstrated that down-regulation of BECN1 by microRNA-9 could enhance the sensitivity of A549 cells to cisplatin and increased cisplatin-induced apoptosis, indicating a pro-survival effects of BECN1 [21]. Here we also detected the role of BECN1 in NSCLC cells. BECN1 was knocked down and cell growth assay was performed in H1299 cells. As can be seen from Figure 1(a), knocking down BECN1 only displayed a slight affection on the proliferation of H1299 cells. Colony formation assay showed that there were no significant effects on the colony forming ability of H1299 cells with BECN1 knockdown (Figure 1(b)). Similar results were obtained in A549 cells with BECN1 knockdown (Figure 1(c–d)). Overexpressing BECN1 also showed no significant effects on the proliferation or colony forming abilities in H1299 and A549 cells (Supplementary Figure 1(a–d)).

### BECN1 expression does not affect cell cycle in NSCLC cells

**Figure 2.**
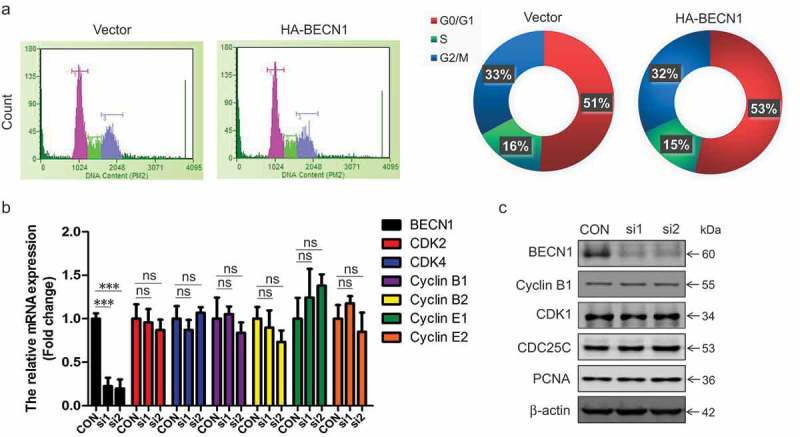
*The cell cycle progression is not affected by BECN1 expression*. (a) H1299 cells were transfected with pCMV-HA or pCMV-HA-BECN1. Forty-eight hours later, cells were harvested and analyzed by flow cytometry (Left panel). The quantification of cell number in each phase of the cell cycle derived from left panel was marked with different colors [red: quiescent phase and the first gap phase (G0/G1 phase), green: DNA synthesis phase (S phase), blue: the second gap phase and mitotic phase (G2/M phase)] (Right panel). (b) The mRNA levels of the cell cycle-related genes were determined by Q-PCR in H1299 cells. Data represent the average of three independent experiments (mean±SD). ***, *P*≤ 0.001; ns, *P >*0.05. (c) The protein levels of the cell cycle-related genes were determined by Western blot using indicated antibodies in H1299 cells transfected with BECN1 siRNAs.

To get further insight into the effects of BECN1 on cell proliferation, we examined the cell cycle progression in H1299 cells overexpressing BECN1. There were no interferences on the cell cycle progression when overexpressing BECN1 in H1299 cells (Figure 2(a)). We also tested the expression of the cell cycle-related genes in H1299 cells with BECN1 knockdown. As expected, there were no obvious changes on the mRNA levels of cell cycle-related genes when knocking down BECN1 (Figure 2(b)). We next examined the protein expression of cell cycle-related gene and there were also no significant changes (Figure 2(c)). From these results, we concluded that the expression of BECN1 did not significantly affect the proliferation of NSCLC cells.

### The expression of BECN1 affects the migration of NSCLC cells

**Figure 3.**
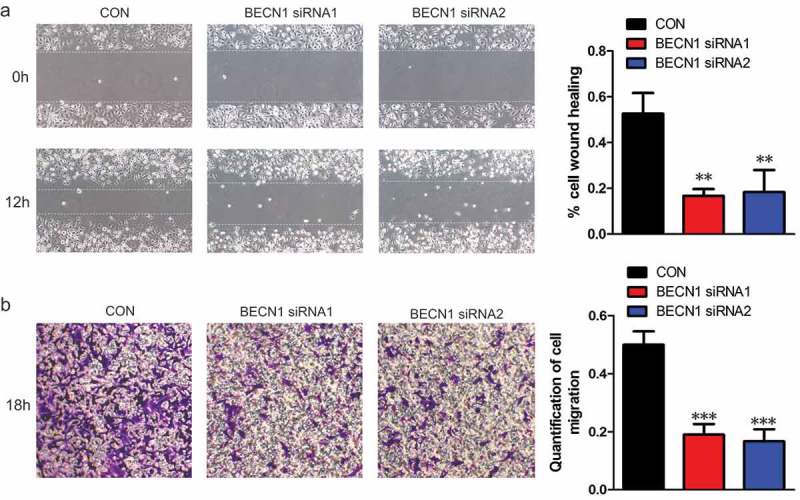
*The expression of BECN1 affects the migratory ability of NSCLC cells*. (a) H1299 cells were transfected with BECN1 siRNAs and wound healing assay was performed. The photographs were taken at 0 and 12 h (Left panels). Cell migration was quantified by measuring the difference in area between the leading edge and the initiation edge of the experiment. The wound area was assessed by the ImageJ software. **, *P*≤ 0.01 (Right panel). (b) BECN1 was knocked down in H1299 cells by specific siRNAs and transwell assay was performed. After 18 h, the migrated cells were fixed and stained with 2% crystal violet. The photographs were taken under 100× magnification (Left panels). The dyes were dissolved by 10% acetic acid and the quantification of cell migration was determined by the absorbance at 595nm. Data represent the average of three independent experiments. ***, *P*≤ 0.001 (Right panel).

**Figure 4.**
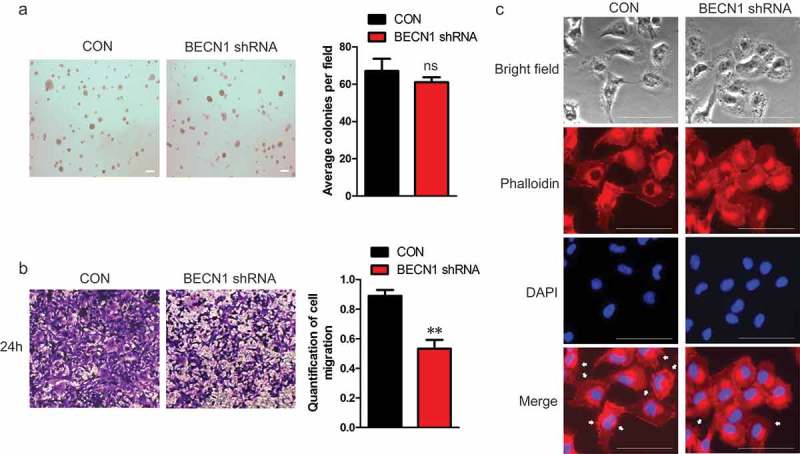
*H1299 cells stably knocking down BECN1 show reduced migratory ability*. (a) H1299 cells stably knocking down BECN1 were used to perform soft agar assay. After two weeks, the Photographs were taken under 40× magnification. Scale bar: 200μm (Left panel). Colonies larger than 50μM were scored. Data represent the average of three independent experiments (mean±SD). ns, *P >*0.05 (Right panel). (b) Transwell assay was performed using H1299 cells stably knocking down BECN1. The migrated cells were fixed and stained with 2% crystal violet. The photographs were taken under 100× magnification. (Left panels). The dyes were dissolved by 10% acetic acid and the quantification of cell migration was determined by the absorbance at 595nm. Data represent the average of three independent experiments. **, *P*≤ 0.01 (Right panel). (c) H1299 stable cell line with BECN1 knockdown were stained with the fluorescent Phalloidin and DAPI. Photographs were taken under 200× magnification using fluorescence microscope. The white arrow indicated filopodium. Scale bar: 50μm.

As BECN1 expression did not affect the proliferation of NSCLC cells, we wanted to know the effects of BECN1 on cell migration. BECN1 was knocked down and wound healing assay was performed. Figure 3(a) shows that knocking down BECN1 apparently slowed down the migratory rate of H1299 cells. To further demonstrate this result, transwell assay was performed. Knocking down BECN1 significantly attenuated the migration of H1299 and A549 cells (Figure 3(b), Supplementary Figure 2(a)). We next overexpressed BECN1 in NSCLC cells and assessed the migratory ability. The cells overexpressing BECN1 migrated much faster than vector control (Supplementary Figure 2(b–c)). We then constructed H1299 stable cell lines with BECN1 knockdown. We purchased four shRNAs from Origene and the knockdown efficiency was examined. Supplementary Figure 3(a) shows that shRNA1 and 3 had better knockdown efficiencies than the others and we used these two shRNAs to construct stable cell lines. Supplementary Figure 3(b) shows that the expressions of BECN1 in the two stable cell lines were much lower than control cell line, indicating that the BECN1-knockdown stable cell lines were constructed successfully. We next detected the proliferation of the stable cell lines. There were no differences in proliferation rate between control cell line and BECN1-knockdown stable cell lines (Supplementary Figure 3(b), bottom panel). To further confirm the effects of BECN1 on NSCLC cell proliferation, soft agar assay was performed. Figure 4(a) shows that there were no significant differences on the colony forming abilities between control and BECN1-knockdown cells. We then performed transwell assay and found that the migratory ability of H1299 cells with BECN1 knockdown was significantly attenuated than control cells (Figure 4(b)). To better understand the impact of BECN1 on cell migration, the actin filaments were stained using the fluorescent Phalloidin to see the formation of filopodium. As shown by fluorescence microscopy, the filopodium in H1299 cells with BECN1 knockdown were significantly reduced when compared with control H1299 cells (Figure 4(c)). Taken together, we concluded that BECN1 affected the migration of NSCLC cells.

### BECN1 affects the ubiquitination of Vimentin

**Figure 5.**
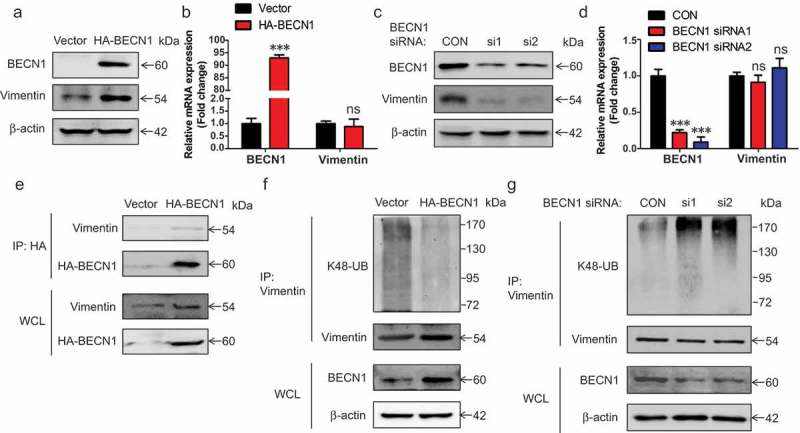
*BECN1 affects the ubiquitination and expression of Vimentin*. (a) H1299 cells were transfected with pCMV-HA-BECN1 plasmid. Forty-eight hours later, Western blot was performed to examine the expression of indicated proteins. (b) H1299 cells were transfected with pCMV-HA-BECN1 plasmid. Forty-eight hours later, total RNA were extracted. The mRNA levels of related genes were determined by Q-PCR. Data represent the average of three independent experiments (mean±SD). ***, *P*≤ 0.001; ns, *P >*0.05. (c) H1299 cells were transfected with BECN1 siRNAs. Forty-eight hours later, the indicated proteins were detected by Western blot. (d) H1299 cells were transfected with BECN1 siRNAs. Forty-eight hours later, total RNA were extracted. The mRNA levels of related genes were determined by Q-PCR. Data represent the average of three independent experiments (mean±SD). ***, *P*≤ 0.001; ns, *P >*0.05. (e) H1299 cells were transfected with pCMV-HA-BECN1 plasmid. Forty-eight hours later, HA-BECN1 was immunoprecipitated using mouse anti-HA antibody followed by Western blot. Vimentin was detected using rabbit anti-Vimentin antibody and HA-BECN1 was detected using rabbit anti-HA antibody. (f) H1299 cells were transfected with pCMV-HA-BECN1 plasmid. Forty-eight hours later, endogenous Vimentin was immunoprecipitated using mouse anti-Vimentin antibody. Western blot was performed to examine the expression of indicated proteins. The K48-linked ubiquitination was detected using K48-linkage specific polyubiquitin antibody. Vimentin was detected using rabbit anti-Vimentin antibody. (g) H1299 cells were transfected with BECN1 siRNAs. Forty-eight hours later, endogenous Vimentin was immunoprecipitated using mouse anti-Vimentin antibody. Western blot was performed to examine the expression of indicated proteins. The K48-linked ubiquitination was detected using K48-linkage specific polyubiquitin antibody. Vimentin was detected using rabbit anti-Vimentin antibody.

The bright field pictures in Figure 4(c) showed that the cell morphologies were different between control and BECN1-knockdown cells. The BECN1-knockdown cells showed round morphology when compared with control cells, indicating that knocking down BECN1 might affect the EMT process. We first examined the expression of EMT markers in BECN1-knockdown stable cell lines. These cells showed reduced protein expression of mesenchymal markers (Vimentin, β-catenin, Snail1, Twist1), while the expression of epithelial marker (E-cadherin) increased (Supplementary Figure 4(a)). These results confirmed that knocking down BECN1 inhibited the EMT process. We also examined the mRNA expression of these EMT markers. The mRNA changes of the EMT markers were consistent with the protein changes except Vimentin (Supplementary Figure 4(b)). The mRNA expression of Vimentin did not change significantly in BECN1-knockdown stable cell lines. This suggested that BECN1 might affected the protein stability of Vimentin. Vimentin is the predominant intermediate filament protein in mesenchymal cells, and is implicated in metastasis and cancer cell migration in breast and colon cancer cell lines [22]. Thus, we focused on the regulation mechanism of BECN1 on the expression of Vimentin. Figure 5(a) shows that overexpression of BECN1 increased the protein expression of Vimentin. Immunofluorescence staining also demonstrated that overexpressing BECN1 increased the intensity of Vimentin staining compared with vector control (Supplementary Figure 5(a)). However, the mRNA level of Vimentin was not affected (Figure 5(b)). Knocking down BECN1 using siRNAs decreased the protein expression of Vimentin and the intensity of Vimentin staining with no influence on Vimentin mRNA level (Figure 5(c–d) and Supplementary Figure 5(b)). These results indicated that BECN1 might affect the expression of Vimentin through post-translational modification. In order to figure out if BECN1 interacted with Vimentin, immunoprecipitation was performed and we found that BECN1 bound with Vimentin (Figure 5(e)). We also detected the colocalization of Vimentin with BECN1 using Immunofluorescence staining. BECN1 (green) and Vimentin (red) were stained with indicated antibodies and the cells in merged the picture showed a yellow color, indicating the colocalization of Vimentin with BECN1 (Supplementary Figure 5(c)). The K48-linked ubiquitination was always associated with proteasome-mediated degradation [23]. We next detected the effects of BECN1 on K48-linked ubiquitination of Vimentin. BECN1 was knocked down or overexpressed in H1299 cells and endogenous Vimentin was immunoprecipitated using specific antibody. We detected K48-linked ubiquitination using an antibody specifically targeting ubiquitin chains that were K48-linked and discovered that overexpressing BECN1 significantly decreased K48-linked ubiquitination of Vimentin, while knocking down BECN1 had an opposite effect (Figure 5(f–g)). These results demonstrated that BECN1 affected the expression of Vimentin through regulating its K48-linked ubiquitination.

### The de-ubiquitination of vimentin by USP14 depends on the expression of BECN1

**Figure 6.**
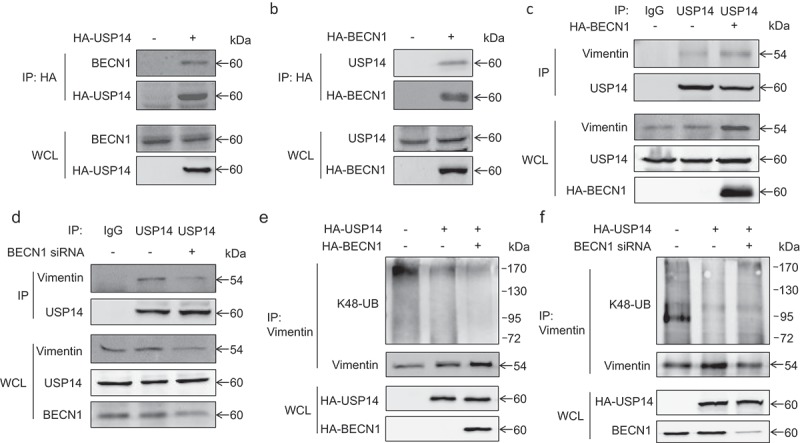
*The de-ubiquitination of Vimentin by USP14 depends on the expression of BECN1*. (a) H1299 cells were transfected with pCMV-HA-USP14 plasmid. Forty-eight hours later, HA-USP14 was immunoprecipitated using rabbit anti-HA antibody followed by Western blot. BECN1 was detected using mouse anti-BECN1 antibody and HA-USP14 was detected using mouse anti-HA antibody. (b) H1299 cells were transfected with pCMV-HA-BECN1 plasmid. Forty-eight hours later, HA-BECN1 was immunoprecipitated using mouse anti-HA antibody followed by Western blot. USP14 was detected using rabbit anti-USP14 antibody and HA-BECN1 was detected using rabbit anti-HA antibody. (c) H1299 cells were transfected with pCMV-HA-BECN1 plasmid. Forty-eight hours later, immunoprecipitation was performed using normal rabbit IgG and rabbit anti-USP14 antibody followed by Western blot. USP14 was detected using mouse anti-USP14 antibody and Vimentin was detected using mouse anti-Vimentin antibody. (d) H1299 cells were transfected with BECN1-specific siRNA. Forty-eight hours later, immunoprecipitation was performed using normal rabbit IgG and rabbit anti-USP14 antibody followed by Western blot. USP14 was detected using mouse anti-USP14 antibody and Vimentin was detected using mouse anti-Vimentin antibody. (e) H1299 cells were transfected with pCMV-HA-USP14 alone or co-transfected with pCMV-HA-USP14 and pCMV-HA-BECN1. Forty-eight hours later, immunoprecipitation was performed using mouse anti-Vimentin antibody. The K48-linked ubiquitination was detected using K48-linkage specific polyubiquitin antibody. Vimentin was detected using rabbit anti-Vimentin antibody. (f) H1299 cells were transfected with pCMV-HA-USP14 alone or co-transfected with pCMV-HA-USP14 and BECN1 siRNA. Forty-eight hours later, immunoprecipitation was performed using mouse anti-Vimentin antibody. The K48-linked ubiquitination was detected using K48-linkage specific polyubiquitin antibody. Vimentin was detected using rabbit anti-Vimentin antibody.

USP14 was reported to regulate the ubiquitination of Vimentin through de-ubiquitination [24]. As BECN1 affected the ubiquitination of Vimentin, we next wondered if this effects was related to USP14. Immunoprecipitation was performed and we found that BECN1 could interact with USP14 (Figure 6(a–b)). What’s more, the interaction between USP14 and Vimentin depended on the expression of BECN1. When we overexpressed BECN1 in H1299 cells, the protein level of Vimentin immunoprecipitated by USP14 was increased (Figure 6(c)). On the contrary, the protein level of Vimentin immunoprecipitated by USP14 was reduced when knocking down BECN1 (Figure 6(d)). We next detected the effects of BECN1 on USP14 mediated de-ubiquitination of Vimentin. Figure 6(e) shows that overexpressing BECN1 could enhanced the de-ubiquitination effects of USP14 on Vimentin, while knocking down BECN1 had an opposite effect (Figure 6(f)). These results demonstrated that BECN1 affected the ubiquitination of Vimentin through regulating the interaction between USP14 and Vimentin.

## Discussion

Autophagy is first associated with cancer through the identification of BECN1 [5]. However, the exact role of BECN1 in cancer progression is still obscure. It was reported that BECN1 was monoallelically deleted in ovarian, breast and prostate cancers [6,7]. However, there was a study demonstrated that the expression of BECN1 was retained in high-grade serous ovarian tumors although a prevalent monoallelic deletion of BECN1 gene was observed in TCGA database [10]. This indicated that monoallelic deletion in BECN1 gene might not fully reflect the protein expression of BECN1 in cancer cells. In NSCLC, previous studies demonstrated that the expression of BECN1 was significantly reduced in tumor tissues [12,13]. However, an interesting phenomenon was that the expression of BECN1 in poorly differentiated NSCLC tissues was higher than in well- and middle-differentiated tissues, indicating that the roles of BECN1 might be different according to tumor malignancy [25]. Previous study demonstrated that knocking down BECN1 in A549 cells promoted cell proliferation. However, this study only performed MTT assay to assess cell proliferation and the relatively large variation attenuated the stringency of the result. In our present study, multiple experiments were used to assess the proliferation of NSCLC cells with BECN1 knockdown or overexpression. We found that modulating the expression of BECN1 did not significantly affect the proliferation of NSCLC cells but had a strong effect on the migration of NSCLC cells. These results prompted us to suppose that BECN1 might have some tumor-promoting effects in NSCLC cells. In fact, a serious of studies have implicated an oncosupportive role of BECN1. In gastric and colorectal cancer, the expression of BECN1 remained a high level in tumor tissues [9]. Metastatic hepatocellular carcinoma cells with suppressed BECN1 expression failed to survive in the metastatic niche [26]. Genetically inhibition of BECN1 blocked tumor formation in mammary cancer stem cells [11]. In BECN1 ± mouse, cell death was increased in tumor hypoxic region [27]. All these results together with our present data indicate that BECN1 functions as an oncogene at least in some kind of cancer cells or under certain circumstance.

Until now, the role of BECN1 in cancer cell migration is still contradictory and the molecular mechanism is not clear. In tongue squamous cell carcinoma, overexpression of BECN1 inhibited cell migration, while knockdown of BECN1 promoted migration [28]. In another study, the ring figure protein 216 (RNF216) promoted colorectal cancer cell migration by enhancing proteasomal degradation of BECN1 [29]. On the other hand, BECN1 was also reported to promote tumor migration. In MDA-MB-231 cells, BECN1-knockout inhibited the migratory and invasive ability by reversing signals of EMT [30]. In hepatomas cells, sphingosine kinase 1 (SPHK1) could induce EMT and promote cell invasion by enhancing the K63-linked ubiquitination of BECN1, thus accelerating CDH1/E-cadherin lysosomal degradation [31]. In our study, we found that BECN1 could interact with Vimentin and affected USP14 mediated de-ubiquitination of Vimentin, thus regulating cell migration. To our knowledge, this is the first study demonstrating that BECN1 could directly interact with migration-associated protein and affect its functions. These findings also indicated that the function of BECN1 was complicated and not limited to autophagy. So more studies need to be done to fully clarify the function of BECN1 in cancer cells. Taken together, our studies demonstrated that BECN1 could promote the migration of NSCLC cells through affecting the ubiquitination and expression of Vimentin. This adds new evidence that BECN1 have oncosupportive roles and provides a new therapeutic strategy that inhibiting the function of BECN1 may be an effective way for the treatment of NSCLC under certain circumstance.

## Materials and methods

### Reagents and plasmids

BECN1 siRNAs were synthesized by Thermo Fisher Scientific. The plasmids pCMV-HA-BECN1 and pCMV-HA-USP14 were constructed by ourselves. Antibody against BECN1 was ordered from OriGene (TA502643). Mouse anti-β-actin antibody was purchased from Proteintech (66009–1-lg). Mouse anti-HA monoclonal antibody was ordered from Thermo Fisher Scientific (26183). Rabbit anti-HA polyclonal antibody was ordered from proteintech (51064–2-AP). K48-linkage specific polyubiquitin antibody was purchased from Cell signaling (8081). Mouse and rabbit anti-Vimentin antibodies were ordered from abcam and proteintech (ab8978, 10366–1-AP). The cell cycle-related genes Cyclin B1, CDK1, CDC25C, PCNA were purchased from proteintech (55004–1-AP, 19532–1-AP, 16485–1-AP, 10205–2-AP). The EMT markers E-cadherin, β-catenin, Snail1, and Twist1 were purchased from proteintech (20874–1-AP, 51067–2-AP, 13099–1-AP, 25465–1-AP). Mouse and rabbit anti-USP14 antibodies were ordered from OriGene and proteintech (TA324906, 14517–1-AP). TRITC-conjugated Goat anti-Rabbit IgG and FITC-conjugated Goat anti-mouse IgG were purchased from proteintech (SA00007-2, SA00003-1).

### Cell culture

The non-small cell lung cancer (NSCLC) cell lines (H1299, A549) were cultured in Roswell Park Memorial Institute 1640 medium (RPMI 1640) (Invitrogen, C11875500BT), containing 10% fetal bovine serum (FBS) (Excel, FCS100). All the cells were incubated at 37℃ with 5% CO_2_.

### Cell growth assay

The cells were plated in 24-well plates at a density of 5000 cells per well in 0.5 ml of RPMI 1640 supplemented with 10% FBS. The medium was changed every other day. At the indicated time, the cells were fixed with 4% formaldehyde and then stained with 2% crystal violet. The dyes were finally dissolved by 10% acetic acid and measured by the absorbance at 595nm.

### Colony formation assay

Colony formation assay was conducted with H1299 cells and A549 cells that were grown in RPMI 1640 with 10% FBS in six-well plates. After transfection, the cells were trypsinized; then, 500 cells were seeded in six-well plates. The cells were then grown for 10 days, with the medium changed every other day. After 10 days, the cells were fixed with 4% formaldehyde and then stained with 2% crystal violet. The images were acquired by a digital camera (Canon, EOS70D).

### Soft agar assay

For soft agar assay, 10^4^ cells were suspended with RPMI 1640 supplemented with 10% FBS and 0.3% agarose followed by plating on the top of a solidified layer of RPMI 1640 supplemented with 0.5% agarose and 10% FBS. Fresh medium with 10% FBS and 0.3% agarose were added to the cells every week. Two weeks later, colonies larger than 50μM were scored.

### Scratch wound healing assay and transwell migration assay

We used scratch wound healing assay and transwell migration assay to measure cell migration. H1299 cells after being treated were plated in six-well plates, when the cells reached a confluent monolayer of 80–90% density, we scraped the monolayer a straight line to create a ‘scratch’ using a 200 μl pipette tip. After debris being washed by phosphate-buffered saline (PBS) and fresh media containing 1% FBS being added, cells were photographed by phase contrast microscope (Olympus IX71).

Transwell migration assay was carried out using 8μm pore size transwell chambers (BD Falcon). After being treated, the cells were trypsinized and counted, 10^5^ cells in 0.2 ml media supplemented with 1% FBS were plated in the upper chamber. Five hundred micro litres RPMI 1640 supplemented with 10% FBS was added in the lower chamber of the transwell device. At the indicated time, cells migrated from the upper chamber to the lower chamber on the membrane were fixed with 4% formaldehyde for 30 min, then stained with 2% crystal violet for 15 min. After washed with 1× PBS to the water clear, the cells were photographed using a light microscope (Olympus IX71).

### Western blot

Cell lysates were subjected to 10% sodium dodecyl sulfate-polyacrylamide gel electrophoresis (SDS-PAGE) and transferred to polyvinylidene fluoride (PVDF) membranes (Milipore). The membranes were blocked with 5% skim milk (BD, 232100) for 1 h at room temperature and incubated with the indicated antibodies overnight at 4°C and detected with horseradish peroxidase-conjugated secondary antibodies. Protein bands were visualized after incubation with Pro-Light chemiluminescence detection kit (TIANGEN, PA112-01) using a digital gel image analysis system TANON 5500.

### Immunoprecipitation

After cells were lysed with Nonidet P 40 (NP-40) lysis buffer containing PMSF (DINGGUO，WB0181) and cocktail, the cell lysates were precleaned with protein G agarose at 4℃ for 1 h, then the supernatant was incubated with indicated antibodies and protein G agarose beads (Roche, 11243233001) at 4℃ overnight. On the second day, immunocomplexes combined with beads were washed by lysis buffer, followed by subjected to Western blot.

### RNA purification and quantitative real-time polymerase chain reaction (Q-PCR) analysis

Total RNA were extracted by TRIzol reagent (Invitrogen, 15596–026). The cDNA was synthesized using PrimeScript RT reagent kit (Takara, RR047A). Q-PCR experiments were performed by SYBR Green Premix Ex Taq II kit (Takara, RR820A) using ABI ViiATM 7 Real-Time PCR System. GAPDH was used as a control.

### Gene overexpression and siRNA knockdown

As to knockdown experiment, the cells were seeded 18–24 h prior to transfection. A nonspecific oligonucleotide from Thermo Fisher Scientific was used as a negative control. The siRNAs were transiently transfected using SuperFectin siRNA Transfection Reagent (Pufei, Shanghai, 2103–100). For gene overexpression, the cells were transfected with indicated plasmids using SuperFectin DNA Transfection Reagent kit (Pufei, Shanghai, 2102–100). Forty-eight hours later, the transfection efficiency was determined by Western blot using relevant antibodies.

### Construction of stable cell line

H1299 cells were transfected with BECN1 shRNAs (OriGene, TF314484). Forty-eight hours later, 1 μg/ml puromycin was added to select the cells. Single colonies were picked up and cultured in the presence of puromycin for two weeks. The knocking down efficiency was determined by Western blot using BECN1 antibody.

### Cell cycle analysis

Cells were harvested and re-suspended in 0.5 ml PBS. Then, 70% alcohol was used to fix the cells on 0–4℃ for at least 2 h. After centrifugation, the sediment was washed with 1× PBS to remove the alcohol and then 2 × 10^5^ cells were collected. We used 400 μl guava cell cycle reagent (Millipore,4700–0160) to re-suspend the cell pellets and incubated at 37℃ for 15 min, the Millipore Guava easyCyte™ flow cytometer (Millipore) was used to analyze cell cycle.

### Immunofluorescence staining

The cells were seeded in 24-well plates and allowed to settle overnight. Then, the cells were washed with 1× PBS and fixed with methanol at room temperature for 20 min. After washed with 1× PBS for three times, 0.1% Triton X-100 in 1× PBS was added and incubated at room temperature for 5 min. The cells were then blocked with 2.5% BSA for 1 h at room temperature, followed by incubating with indicated antibodies for 2 h at room temperature. After rinsed with 1× PBS, the cells were incubated with indicated secondary antibody for 1 h at room temperature in the dark. Then, the cells were washed with 1× PBS for three times and added with DAPI Fluoromount-G mounting medium (SouthernBiotech, 0100–20). For F-actin staining, the cells were stained by TRITC-Phalloidin (Yeasen, 40734ES75). The cell nuclei were stained by DAPI Fluoromount-G mounting medium. Immunofluorescence photographs were captured with Olympus IX83 inverted microscope and processed with Olympus CellSens™ Microscope Imaging Software.

### Statistical analysis

Data are presented as means ± SD. One-way ANOVA and unpaired t-test were used to make the statistical comparisons, *P*-values≤0.05 were considered to be statistically significant.
