# Supplementary material for: BECN1 promotes the migration of NSCLC cells through regulating the ubiquitination of Vimentin
Source: Cell Adh Migr. 2019 Jul 5;13(1):249–59. doi: 10.1080/19336918.2019.1638690 (PMC6629178; doi:10.1080/19336918.2019.1638690)
Supplement: Supplemental Material [file kcam-13-01-1638690-s001.zip › Supplementary Figure 1.pptx]

## Slide 1
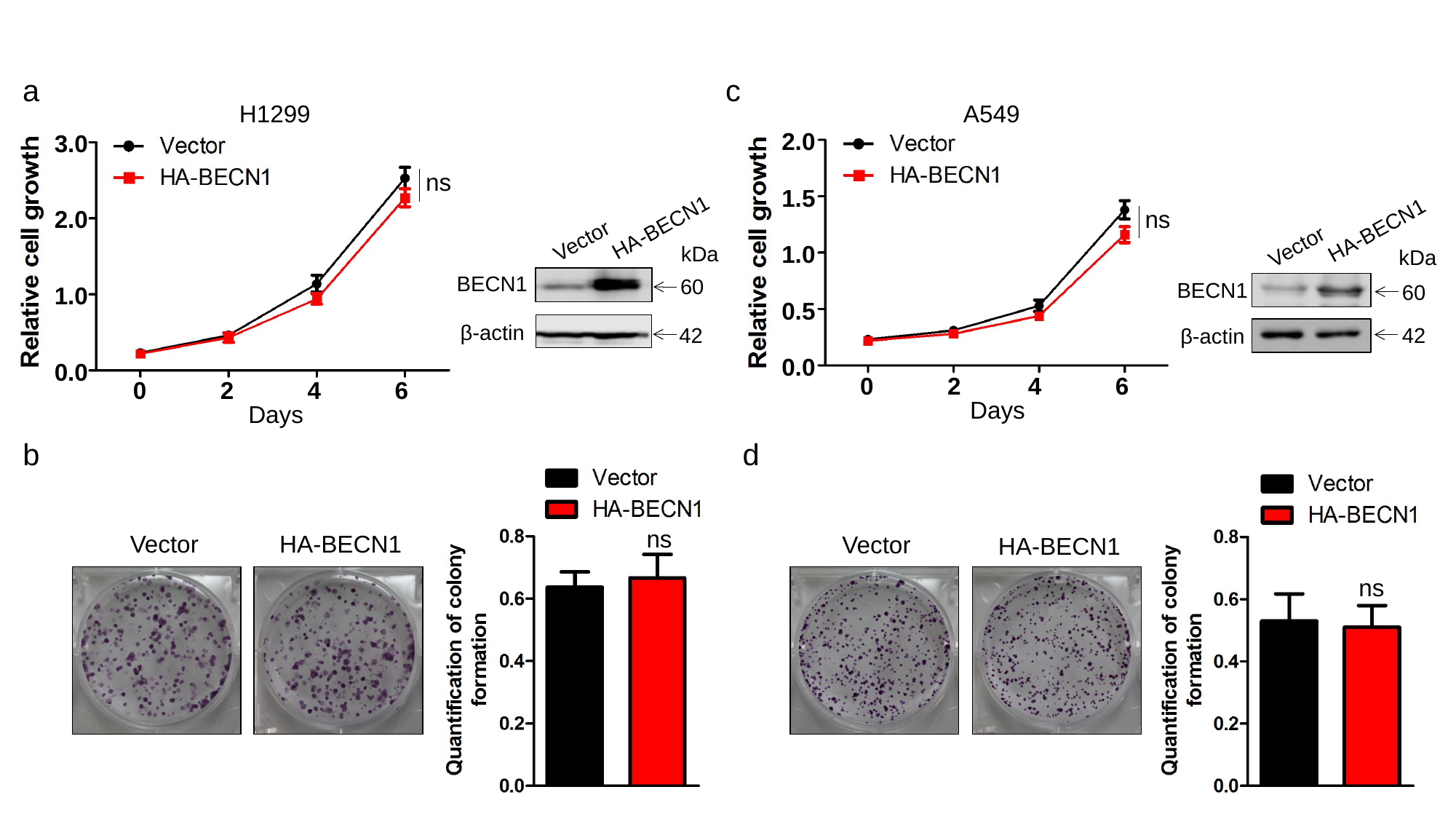

a c
H1299
A549
2.0
1.5
ns
1.0
0.5
0.0
0 2 4 6
3.0
ns
2.0
1.0
0.0
0 2 4 6
HA-BECN1
HA-BECN1
Vector
Vector
kDa
kDa
BECN1
60
BECN1
60
β-actin
42
42
β-actin
Days
Days
b d
ns
Vector
HA-BECN1
Vector
HA-BECN1
ns
