# Supplementary figures and images for: BECN1 promotes the migration of NSCLC cells through regulating the ubiquitination of Vimentin
Source: Cell Adh Migr. 2019 Jul 5;13(1):249–59. doi: 10.1080/19336918.2019.1638690 (PMC6629178; doi:10.1080/19336918.2019.1638690)

## Slide 1
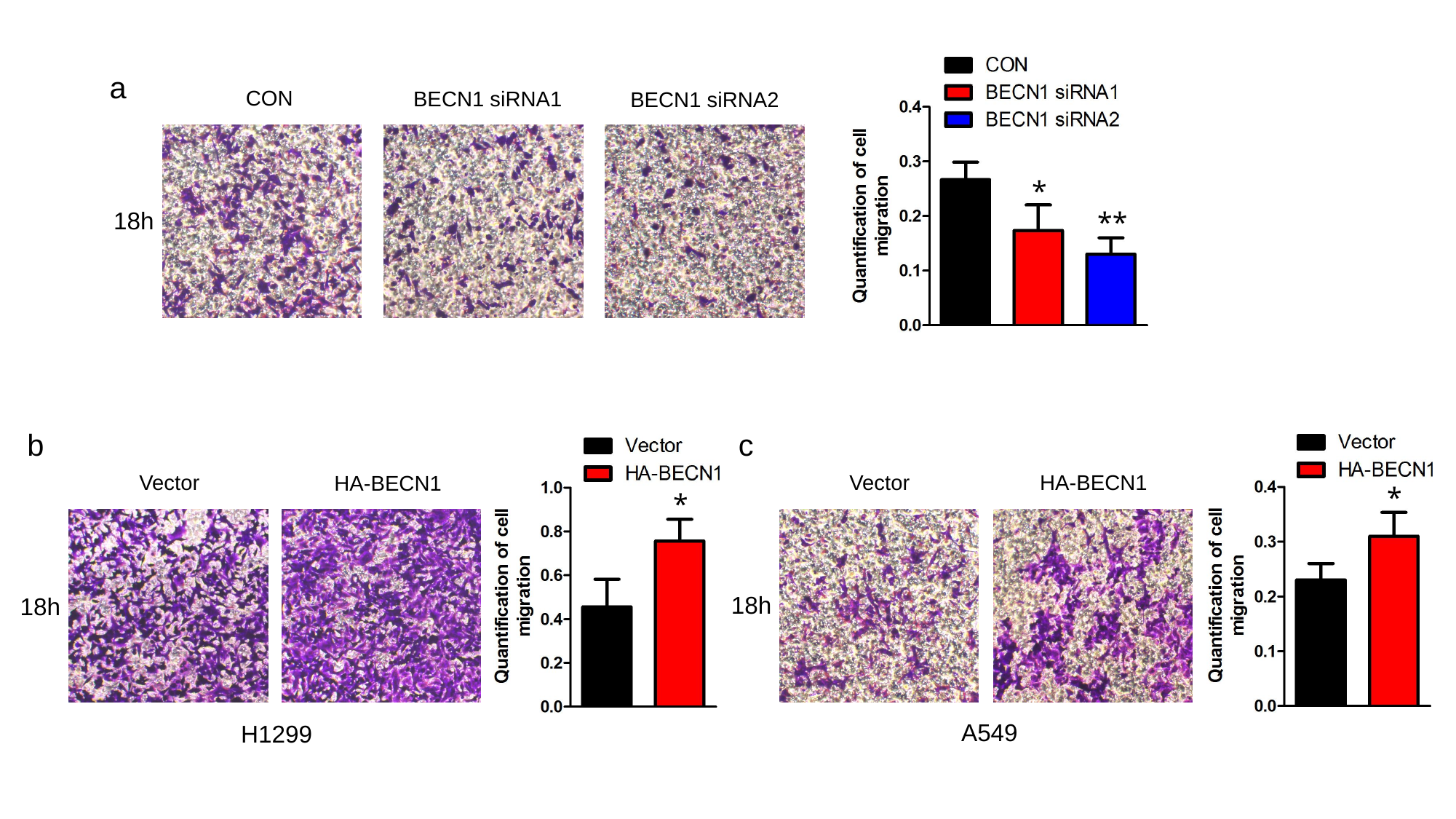

*
**
a
CON
BECN1 siRNA1
BECN1 siRNA2
18h
b c
*
*
Vector
Vector
HA-BECN1
HA-BECN1
18h
18h
A549
H1299

Supplement: Supplemental Material [file kcam-13-01-1638690-s001.zip › Supplementary Figure 2.pptx]

## Slide 1
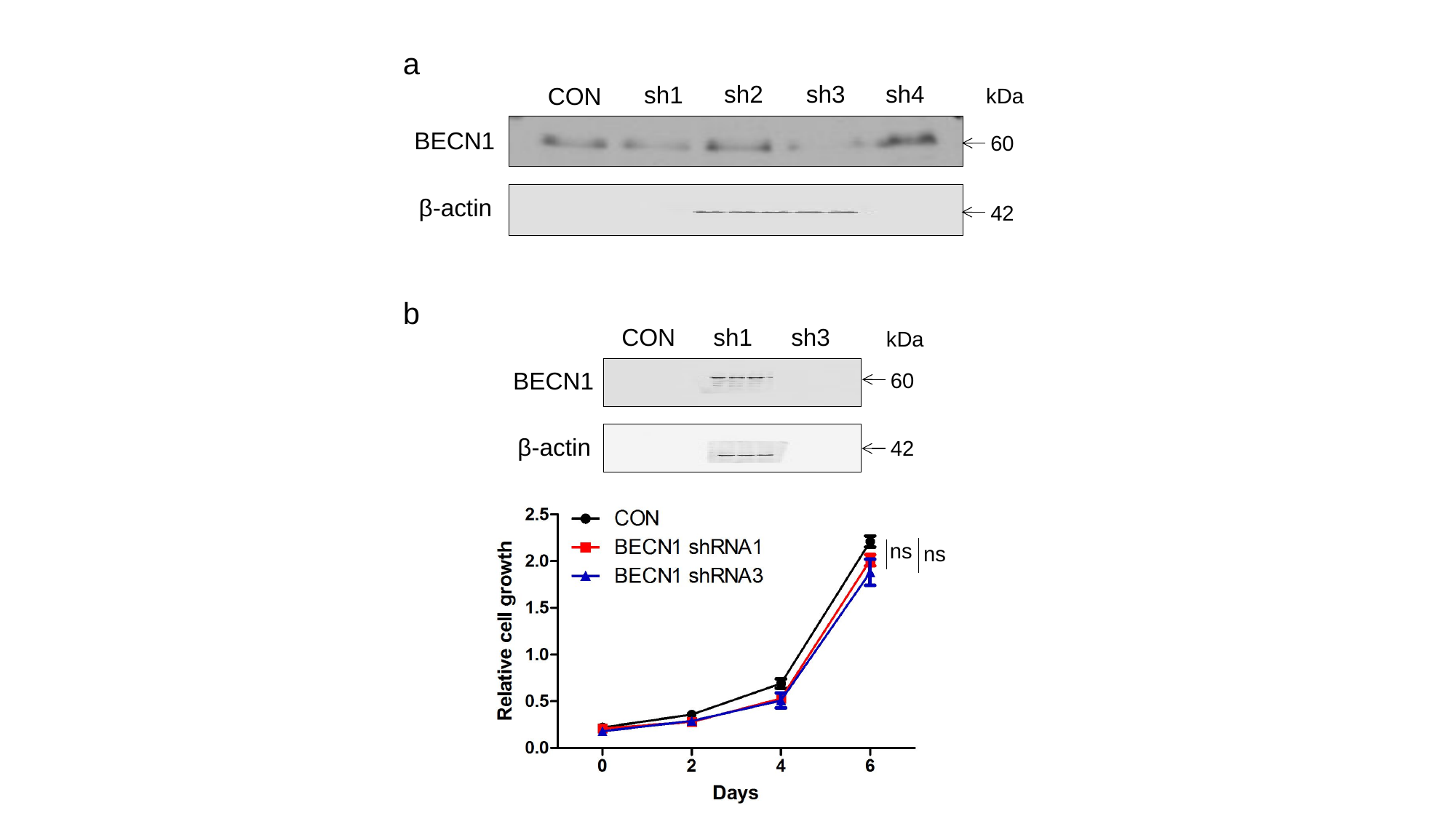

a
sh2
sh3
sh4
sh1
CON
kDa
BECN1
60
β-actin
42
b
CON
sh1
sh3
BECN1
β-actin
kDa
60
42
ns
ns

Supplement: Supplemental Material [file kcam-13-01-1638690-s001.zip › Supplementary Figure 3.pptx]

## Slide 1
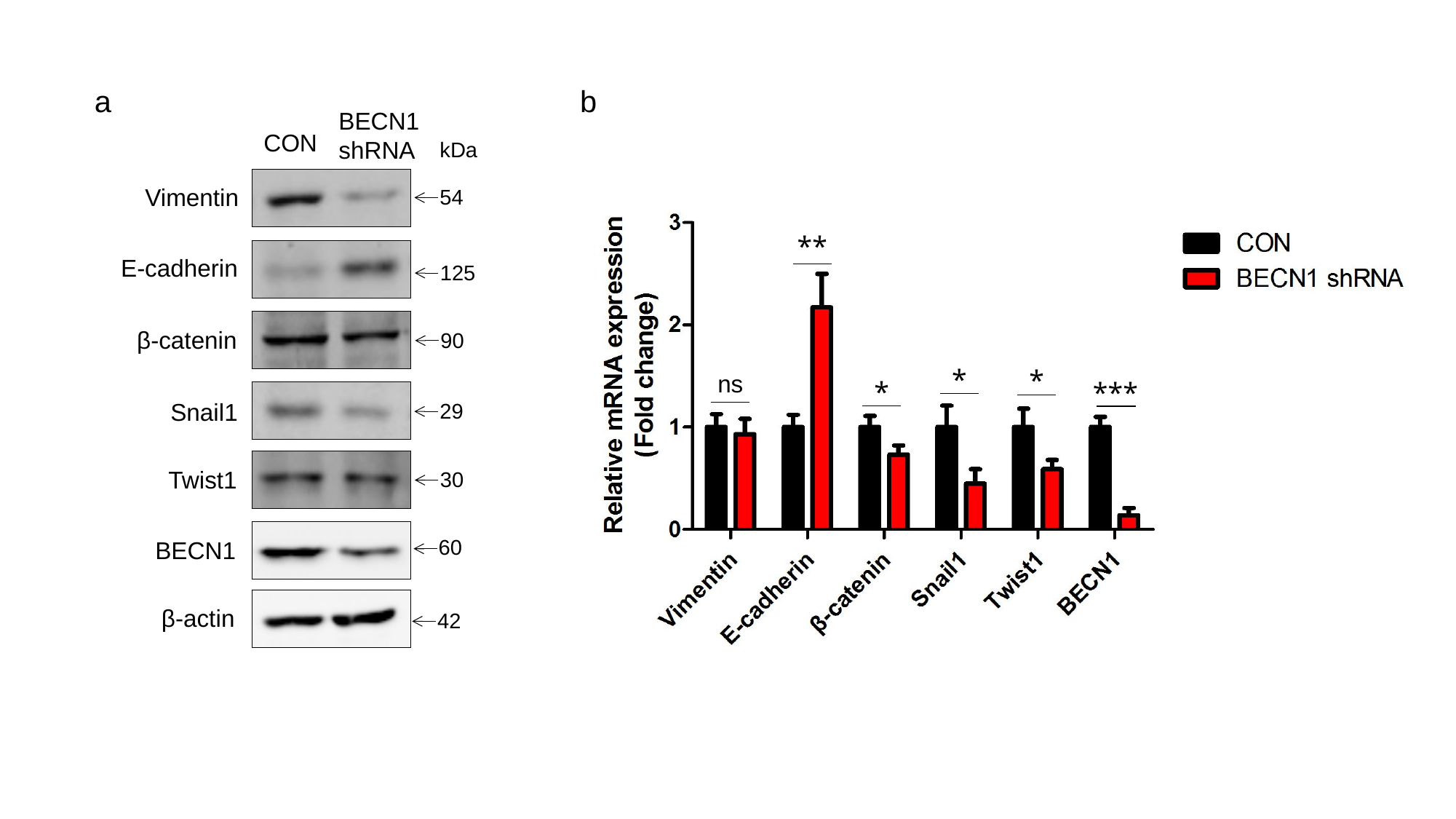

a b
BECN1
shRNA
CON
Vimentin
E-cadherin
β-catenin
Snail1
Twist1
BECN1
β-actin
kDa
54
**
*
*
ns
*
***
125
90
29
30
60
42

Supplement: Supplemental Material [file kcam-13-01-1638690-s001.zip › Supplementary Figure 4.pptx]

## Slide 1
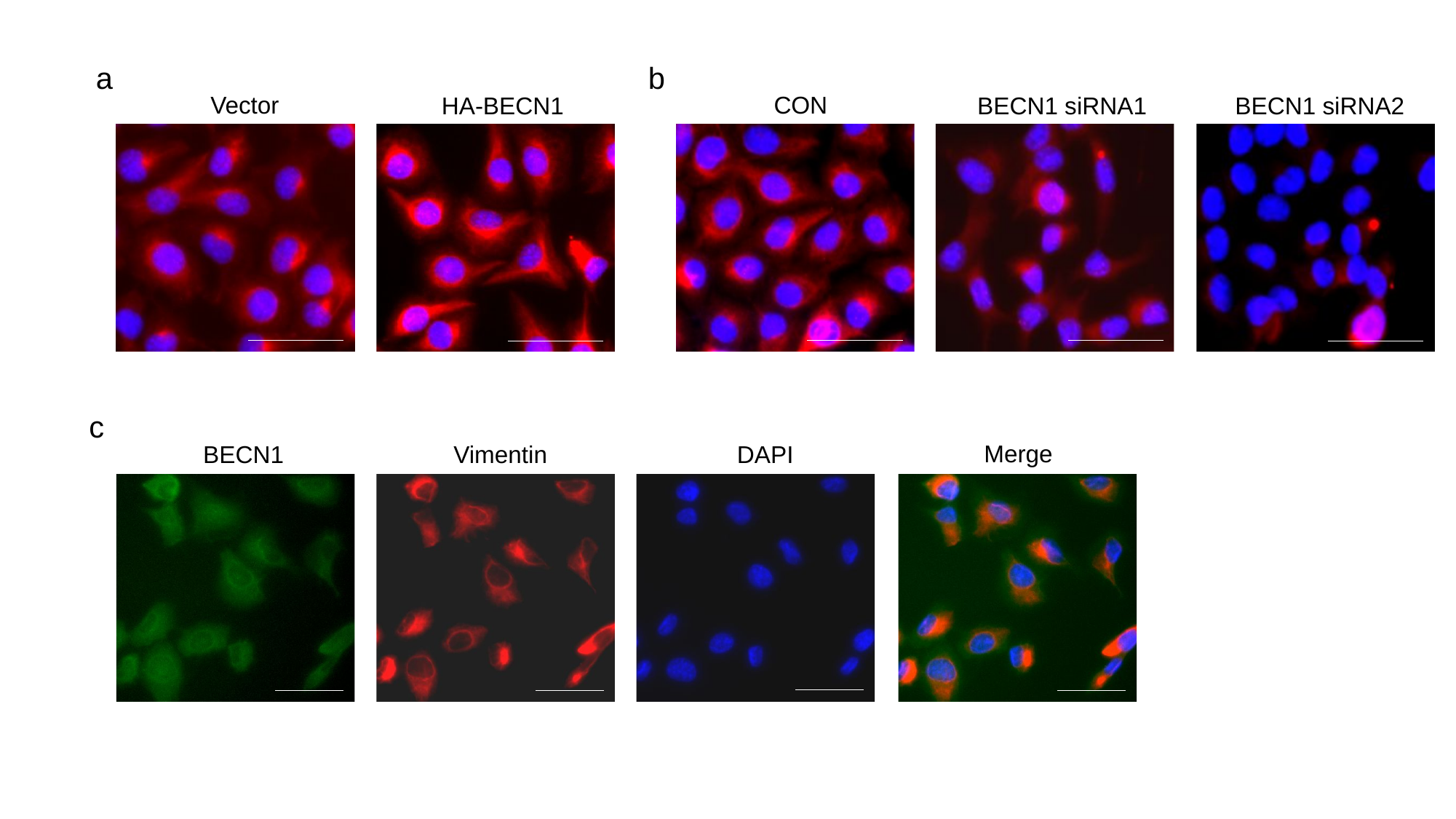

a b
Vector
CON
HA-BECN1
BECN1 siRNA1
BECN1 siRNA2
c
Merge
BECN1
DAPI
Vimentin

Supplement: Supplemental Material [file kcam-13-01-1638690-s001.zip › Supplementary Figure 5.pptx]
