# Supplementary material for: BECN1 promotes the migration of NSCLC cells through regulating the ubiquitination of Vimentin
Source: Cell Adh Migr. 2019 Jul 5;13(1):249–59. doi: 10.1080/19336918.2019.1638690 (PMC6629178; doi:10.1080/19336918.2019.1638690)
Supplement: Supplemental Material [file kcam-13-01-1638690-s001.zip › Supplementary information.doc]

**Supplementary information**

**Supplementary Fig. 1.** *Overexpression of BECN1 does not significantly affect the proliferation of NSCLC cells.* (a) H1299 cells were transfected with vector control or pCMV-HA-BECN1. 24 hours later, cell growth assay was performed. At the indicated times, cells were fixed with 4% formaldehyde and then stained with 2% crystal violet. The dyes were finally dissolved by 10% acetic acid and the relative proliferation was determined by the absorbance at 595nm. Data represent the average of three independent experiments. ns, *P>*0.05 (Left panel). The overexpression efficiency of HA-BECN1 was determined by Western blot using anti-BECN1 antibody (Right panel). (b) H1299 cells were transfected with vector control or pCMV-HA-BECN1 for 24 hours and colony formation assay was performed. After ten days, the cells were fixed with 4% formaldehyde and then stained with 2% crystal violet. The colonies were photographed (Left panel). The dyes were dissolved by 10% acetic acid and the quantification of colony formation was determined by the absorbance at 595nm. Data represent the average of three independent experiments. ns, *P>*0.05 (Right panel). (c) A549 cells were transfected with vector control or pCMV-HA-BECN1. 24 hours later, cell growth assay was performed. At the indicated times, cells were fixed with 4% formaldehyde and then stained with 2% crystal violet. The dyes were finally dissolved by 10% acetic acid and the relative proliferation was determined by the absorbance at 595nm. Data represent the average of three independent experiments. ns, *P>*0.05 (Left panel). The overexpression efficiency of HA-BECN1 was determined by Western blot using anti-BECN1 antibody (Right panel). (d) A549 cells were transfected with vector control or pCMV-HA-BECN1 for 24 hours and colony formation assay was performed. After ten days, the cells were fixed with 4% formaldehyde and then stained with 2% crystal violet. The colonies were photographed (Left panel). The dyes were dissolved by 10% acetic acid and the quantification of colony formation was determined by the absorbance at 595nm. Data represent the average of three independent experiments. ns, *P>*0.05 (Right panel).

**Supplementary Fig. 2.** *BECN1 expression affects the migratory ability of NSCLC cells.* (a) BECN1 was knocked down in A549 cells by specific siRNAs and transwell assay was performed. After 18 hours, the migrated cells were fixed and stained with 2% crystal violet. The photographs were taken under 100× magnification (Left panels). The dyes were dissolved by 10% acetic acid and the quantification of cell migration was determined by the absorbance at 595nm. Data represent the average of three independent experiments. *, *P*≤0.05; **, *P*≤0.01 (Right panel). (b and c) BECN1 was overexpressed in H1299 cells (b) or A549 cells (c) by transfecting pCMV-HA-BECN1 plasmid and transwell assay was performed. After 18 hours, the migrated cells were fixed and stained with 2% crystal violet. The photographs were taken under 100× magnification (Left panels). The dyes were dissolved by 10% acetic acid and the quantification of cell migration was determined by the absorbance at 595nm. Data represent the average of three independent experiments. *, *P*≤0.05 (Right panel).

**Supplementary Fig. 3.** *The proliferation rates of BECN1-knockdown stable cell lines do not show significant differences compared with control cell line.* (a) H1299 cells were transfected with BECN1 shRNAs. 48 hours later, Western blot was performed to detect the knocking down efficiency. (b) The expression of BECN1 in H1299 stable cell lines was detected using Western blot (Top panel). H1299 cells stably knocking down BECN1 were used to perform cell growth assay. At the indicated times, cells were fixed and stained with 2% crystal violet. The dyes were finally dissolved by 10% acetic acid and the relative proliferation was determined by the absorbance at 595nm. Data represent the average of three independent experiments. ns, *P>*0.05 (Bottom panel).

**Supplementary Fig. 4.** *Knocking down BECN1 inhibits the EMT process.* (a) The expression of EMT markers were detected using Western blot in control H1299 cells and BECN1-knockdown stable cell line. (b) The mRNA levels of EMT markers in control H1299 cells and BECN1-knockdown stable cell line were detected by Q-PCR. Data represent the average of three independent experiments (mean±SD). *, *P*≤0.05; **, *P*≤0.01; ***, *P*≤0.001; ns, *P>*0.05.

**Supplementary Fig. 5.** *BECN1 colocalizes with Vimentin and affects its expression.* (a) H1299 cells were transfected with vector control or pCMV-HA-BECN1 for 48 hours. Then, the cells were fixed and stained with anti-Vimentin antibody (red) and DAPI (blue). Photographs were taken under 200×magnification using fluorescence microscope. Scale bar: 50μm. (b) H1299 cells were transfected with control siRNA or BECN1 siRNAs for 48 hours. Then, the cells were fixed and stained with anti-Vimentin antibody (red) and DAPI (blue). Photographs were taken under 200×magnification using fluorescence microscope. Scale bar: 50μm. (c) H1299 cells were fixed and stained with anti-BECN1 antibody (green), anti-Vimentin antibody (red) and DAPI (blue). Photographs were taken under 200× magnification using fluorescence microscope and merged using CellSens software. Scale bar: 50μm.
